# *Rhododendron molle* G. Don Extract Induces Apoptosis and Inhibits Migration in Human Colorectal Cancer Cells and Potential Anticancer Components Analysis

**DOI:** 10.3390/molecules26102990

**Published:** 2021-05-18

**Authors:** Luye Zong, Yan Yang, Jin Zhang, Liangfang Dai, Yuqiang Luo, Jing Yang, Xiangdong Luo

**Affiliations:** College of Life Science, Jiangxi Normal University, Nanchang 330022, China; lyzong119@163.com (L.Z.); Yang0218yan@163.com (Y.Y.); jzhanglj@163.com (J.Z.); qq2367140397@163.com (Y.L.); yj2335258062@163.com (J.Y.)

**Keywords:** *Rhododendron molle* G. Don, HT-29, apoptosis, cell cycle, Bcl-2, GC-MS

## Abstract

*Rhododendron molle* G. Don is one example of traditional Chinese medicine with important medicinal value. In this study, the effects of methanol extract of *R. molle* leaves (RLE) on colorectal cancer HT-29 cells and its potential molecular mechanism were investigated. MTT analysis showed that RLE could significantly inhibit the cell viability and migration of HT-29 cells in a concentration-dependent manner. Cell cycle analyses via flow cytometer suggested that RLE induced DNA fragmentation, indicative of apoptosis, and arrest at the S phase in HT-29 cells. Quantitative real-time PCR (qRT-PCR) analysis showed that RLE could upregulate the mRNA expression of *p53* and *p21* in HT-29 cells, which would result in HT-29 cells being blocked in S phase. Meanwhile, RLE could upregulate the expression of *Bax,* and downregulate the expression of *Bcl-2*, which would induce cell apoptosis. Further western blot analysis showed that the protein expression changes of Bax and P53 were basically consistent with the results of qRT-PCR. In addition, GC-MS analysis detected 17 potential anticancer components in *R. molle.* These results indicate that *R. molle* has significant anticancer activity, which provides some useful information for further study and clinical application for *R. molle*.

## 1. Introduction

Colorectal cancer is a common malignant tumor, and is one of the most common gastrointestinal tumors in the world. Currently, the incidence of colorectal cancer (6.1%) was ranked as the fourth among cancer-related diseases. In addition, the mortality rate was ranked as second at 9.2% [1]. With the change of national diet structure and the improvement of living standards, the incidence and mortality of colorectal cancer in the world has been increasing year by year [2]. At present, surgery, adjuvant radiotherapy, and chemotherapy are the main treatments for colorectal cancer. However, tumor metastases and invasion cause a lower five-year survival rate for colorectal cancer patients of only about 60% [3]. Moreover, the side effects of chemotherapy are great, which brings great psychological and economic pressure to patients [4,5]. Therefore, it is of great significance to develop more effective treatment methods to reduce the mortality of colon cancer. Among them, discovery of new anticancer drugs from natural products is one of the most effective ways.

Previous studies suggested that more than 50% of new anticancer drugs were developed from natural compounds [6,7,8], which provides extensive prospects for developing the new therapeutic agents for colorectal cancer. Many natural products with anticancer activity were able to inhibit the proliferation of cancer cells by intoxicating, apoptosis, blocking its period, or damaging its skeleton [7,9]. For example, the crude extract of *Hedyotis diffusa* blocks cancer cells in the S phase of its period so as to inhibit the proliferation of the cells [10]; *Eleutherococcus giraldii* induces apoptosis of cancer cells by raising the expression of tumor suppressor gene *Bax* and downregulating the expression of *Bcl-2* in the cells [11]. Moreover, green barley leaf extract induced selective antiproliferative and pro-apoptotic activity on leukemia/lymphoma cells, as evidenced by phosphatidylserine externalization, enhanced release of TNF-α, caspase-8, and caspase-3 activation, PARP-1 cleavage, and DNA fragmentation [12]. At the same time, the development and application of anticancer drugs have gradually shifted from traditional drugs with a single target and strong toxicity and side effects to natural drugs with multi-target, multi-level, and multi-directional regulation and less toxicity. Natural compounds have been used to treat various diseases, often combined with anticancer drugs to treat cancer [13,14]. Thus, natural compounds have great potential in the development of new anticancer drugs.

As a traditional Chinese medicine which has been used for thousands of years, *R. molle* has the functions of dispelling wind, removing dampness, and relieving pain, and in modern medicine, it is also increasingly being used clinically [11]. It has been used as an effective drug in the treatment of rheumatoid inflammation, hypertension, narcotic analgesics, chronic bronchitis, dermatophytes, and other diseases [14,15,16]. Moreover, *R. molle* has been widely used as an insecticide, which has stomach toxicity, contact killing, antifeedant, fumigation, and growth inhibition effects on a variety of pests. Modern research shows that *R. molle* contains a lot of diterpenoids, flavonoids, triterpenoids, lignans, and other compounds [16]. Pharmacological studies have confirmed that diterpenoids are effective components with anti-inflammatory, analgesic, and other pharmacological effects. The representative diterpenoid component rhodojaponin III, also known as rhomotoxin, can significantly slow down the heart rate and reduce blood pressure, which can be used for the treatment of supraventricular tachycardia and hypertension [17]. Moreover, *R. molle* is a poisonous plant, and the diterpenes are the major toxic components [18,19]. Our previous studies also showed that *R. molle* has significant anti-inflammatory and antioxidant activities; the methanol extract of *R. molle* leaf could significantly concentration-dependently inhibit NO production and the expression of many proinflammatory cytokines and inflammatory-related enzymes [20].

Previous studies showed that the resources of *R. molle* have been sharply decreased. The medicinal materials supply of *R. molle* was greatly limited [16]. Since the leaves of *R. molle* also have great medicinal value (including anti-inflammatory and antioxidant activities), it may be a good substitute for the roots, flowers, and fruits of *R. molle*. At present, the anticancer activity and mechanism of *R. molle* has not been reported, which restricted the investigation and potential applications of *R. molle*. Therefore, in this present study, the detection system of anticancer activity in vitro was constructed with HT-29 cells as the research object to investigate the effect of RLE on apoptosis and cycle. Furthermore, the potential molecular mechanism of RLE inhibiting the proliferation of colorectal cancer cells was revealed. Additionally, GC-MS was used to analyze the chemical components in RLE and reveal its potential anticancer components. This study would provide some supplementary evidences for the anticancer effect and molecular mechanism of *R. molle*, and it could also establish a foundation for further study and clinical application.

## 2. Results

### 2.1. The Effect of R. molle Extract on Cell Viability

**Figure 1:**
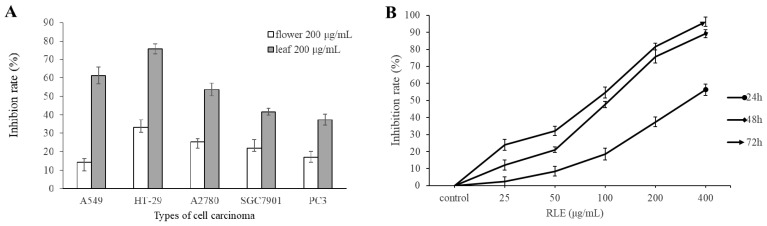
(**A**) Inhibitory effect of RFE and RLE on the growth of five cancer cells at 200 μg/mL for 48 h (`x ± SD, *n* = 3); (**B**) Inhibitory effect of RLE on HT-29 cells (`x ± SD, *n* = 3).

In order to investigate the inhibitory effect of the methanol extract of *R. molle* leaves (RLE) and the methanol extracts from *R. molle* flower (RFE) on the proliferation of cancer cells for 48 h, five different cancer cells (A549, HT-29, A2780, SGC7901, and PC3) were studied by the MTT method in this study. The results showed that the viability of all of the five kinds of cancer cell could be significantly inhibited by RLE (Figure 1A). The IC_50_ values of RLE and RFE on cytotoxicity are shown in Table 1. At the concentration of 200 μg/mL, the inhibition rates of RLE on the proliferation of five kinds of cancer cell (A549, HT-29, A2780, SGC7901, and PC3) were 61.25%, 75.68%, 53.82%, 41.62%, and 37.41%, respectively, and the inhibition rate on HT-29 cells was the highest. However, RFE (extracts from the flower) had little inhibitory effect on the cell viability of the five kinds of cancer cells. When the concentration of RFE was 200 μg/mL, the highest inhibitory rate on HT-29 cells was 33%, and the inhibition rates of the other four kinds of cancer cells were lower than 25%. Therefore, HT-29 cells and RLE were selected for further study. The results showed that the inhibition rate of RLE on HT-29 cell proliferation was dose and time dependent (Figure 1B). When the concentration of 200 μg/mL RLE was used to treat HT-29 cells for 24 h, 48 h, and 72 h, the cell proliferation inhibition rates were 37.41%, 75.69%, and 81.65%, respectively.

### 2.2. The Effect of RLE on Colony Formation of HT-29 Cells

**Figure 2:**
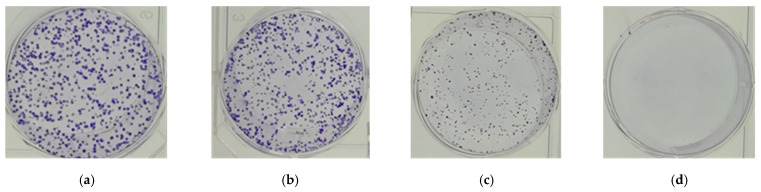
(**a**) Control; (**b**) 50 μg/mL; (**c**) 100 μg/mL; (**d**) 200 μg/mL. The effect of RLE on the colony formation rate of HT-29 cells.

In this study, the colony formation assay was used to determine the long-term effect of the RLE on cell survival rate. HT-29 cells were treated with different concentrations of RLE, and the formation of cell clones was detected after 13 days. The results showed that the colony formation ability of HT-29 cells would be diminished for the RLE treatment (Figure 2). When the concentration was 100 μg/mL, the colony formation of HT-29 cells was significantly reduced. In addition, when the concentration was 200 μg/mL, there was no cell colony formation. These data suggested that RLE treatment generated an irreversible inhibition on cell growth, and that RLE could be a potential antitumor compound.

### 2.3. The Effect of RLE on HT-29 Cell Migration

**Figure 3:**
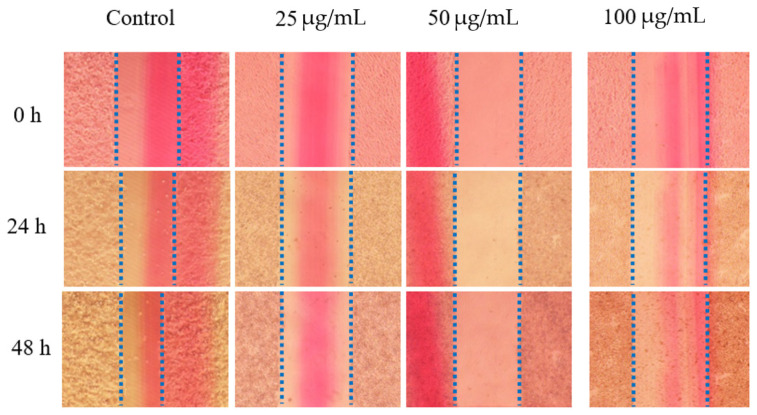
Effect of RLE on the migration of HT-29 cells by wound healing assay.

**Figure 4:**
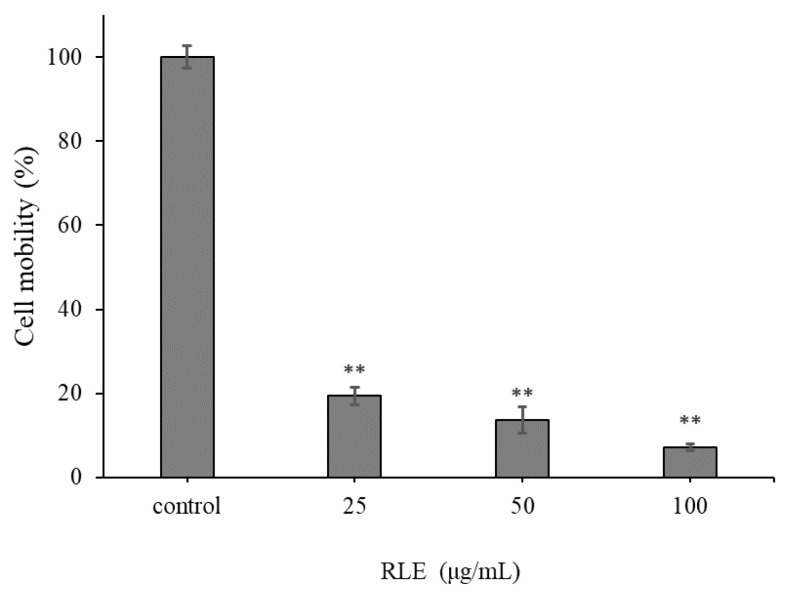
The effect of RLE on the migration of HT-29 cells (`x ± SD, *n* = 3), ** *p* < 0.01 vs. control group, the same below.

HT-29 cells were treated with different concentrations of RLE to detect the effect on cell migration. The results are demonstrated in Figure 3. For RLE treatment, HT-29 cells exhibited decreased migration compared to those in the control group. In addition, the migration rate decreased with the increase of RLE concentration. When the concentration reached 100 μg/mL, the migration rate of HT-29 cells was only 7.15% of that of the control group (Figure 4). The results suggested that RLE could obviously inhibit the migration of cells.

### 2.4. The Effects of RLE on Apoptosis and Cell Cycle of HT-29 Cells

**Figure 5:**
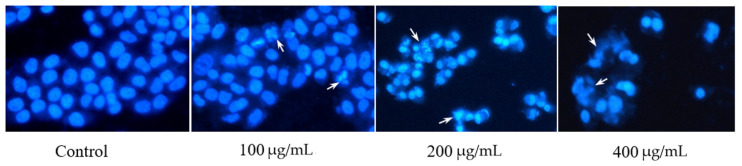
RLE-induced morphological changes on HT-29 cells after 24 h of exposure, analyzed in fluorescence microscope images.

**Figure 6:**
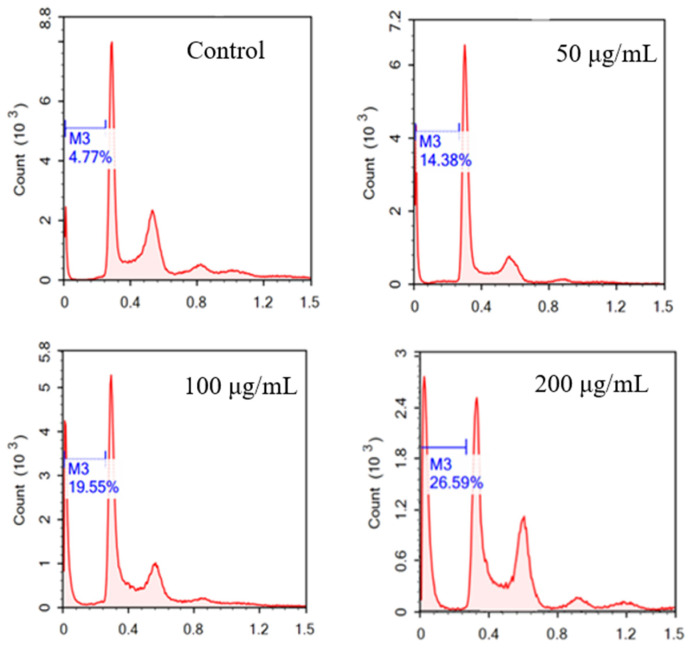
The effect of RLE on the cell cycle distribution of HT-29 cells by flow cytometry.

The nuclear morphology was investigated by DAPI staining after RLE treatment for 24 h. From Figure 5, we can find that the nucleus of HT-29 cells in the control group showed uniform and soft blue fluorescence, and the nuclei were large and oval. After treatment with RLE for 24 h, HT-29 cells showed typical apoptosis. The nucleus showed obvious deformation, chromatin condensation, edge aggregation, crescent, and fluorescent fragments. With the increase in the concentration of the RLE, the change was more obvious. The apoptosis of HT-29 cells was detected by flow cytometry (Figure 6). The results showed that the late apoptotic rate of HT-29 cells was to 4.77%, 14.38%, 19.55%, and 26.59% respectively when treated with 0, 50, 100, and 200 μg/mL RLE for 24 h. The results of the flow cytometry are consistent with those of the DAPI staining method. This suggested that the inhibition of cell proliferation by RLE was due to the promotion of apoptosis in a concentration-dependent manner.

**Figure 7:**
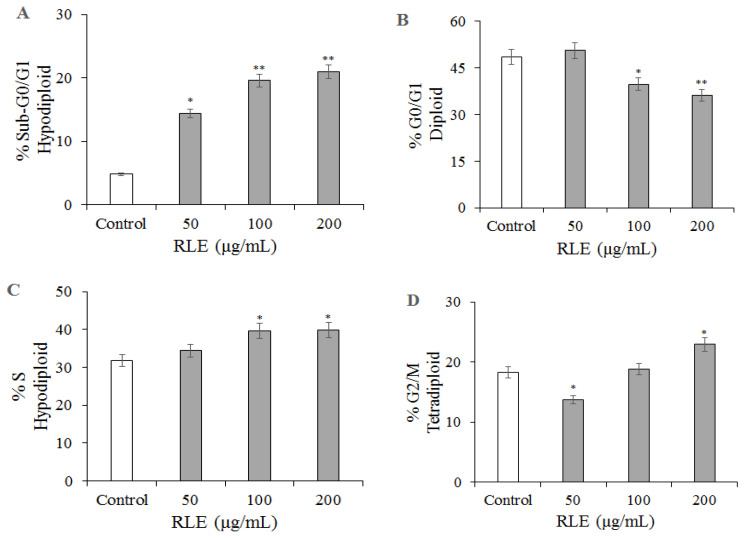
HT-29 cells were treated with different concentrations of RLE, fixed with 70% ethanol, stained with PI, and cell cycle was detected by flow cytometry. (**A**–**D**) The percentages of cells in each phase are plotted along the *y*-axis and the different treatments are graphed along the *x*-axis. (`x ± SD, *n* = 3). * *p* < 0.05 ** *p* < 0.01 vs. control group.

In order to better elucidate the mechanism of apoptosis of HT-29 cell induced by RLE, flow cytometry analysis was used to further investigate the effect of RLE on the HT-29 cell cycle. Results of the cell cycle indicated that cell percentage in the S phase was significantly increased by RLE, but the cells in the G2 and G1 phases were increased (Figure 7). The results showed that 48.60% of HT-29 cells in the control group were in the G1 phase and 31.83% were in the S phase. Compared with the control group, when the concentration was 200 μg/mL, the number of cells in G1 phase decreased to 36.19%, and the number of cells in S phase increased to 39.80%; these findings indicated that treatment with RLE induced remarkable S phase arrest of HT-29 cells.

### 2.5. The Effect of RLE on the Apoptosis-Related Gene Expression

**Figure 8:**
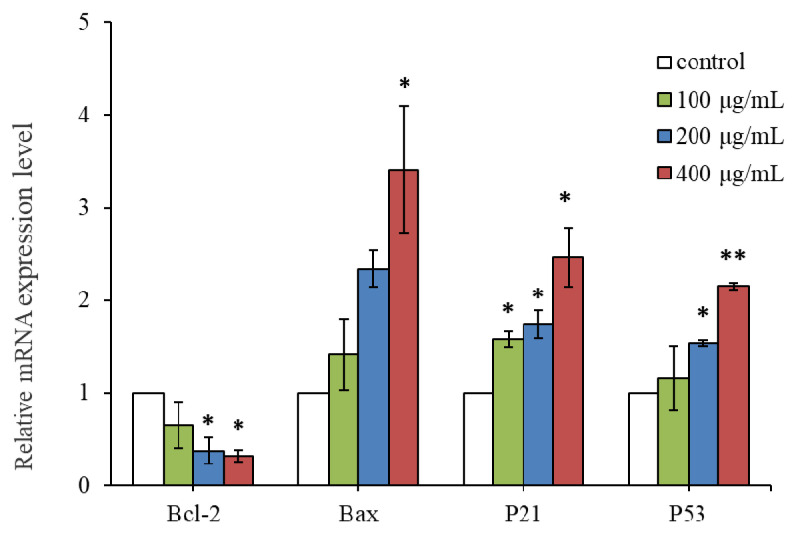
Effect of RLE on expression of cell cycle and apoptosis-associated genes on HT-29 cells (`x ± SD, *n* = 3). * *p* < 0.05 ** *p* < 0.01 vs. control group.

In order to further illuminate the potential anti-cancer mechanism of RLE, qRT-PCR analysis was used to detect the gene expression level of *Bax*, *Bcl-2*, *P21*, and *P53* genes in HT-29 cells (Figure 8). Compared with the control group, RLE would downregulate the expression level of *Bcl-2* in HT-29 cells with dose dependence. When the cells were treated with 200 μg/mL RLE, the gene expression level of *Bcl-2* was only 0.37 times that of the control group. By contrast, RLE would upregulate the expression level of *Bax*, *P21*, and *P53* in HT-29 cells with dose dependence. When the cells were treated with 200 μg/mL RLE, the expression of *Bax*, *P53*, and *P21* genes was upregulated by 2.33, 1.73, and 1.53 times respectively.

### 2.6. The Effects of RLE on the Apoptosis-Related Proteins Expression

**Figure 9:**
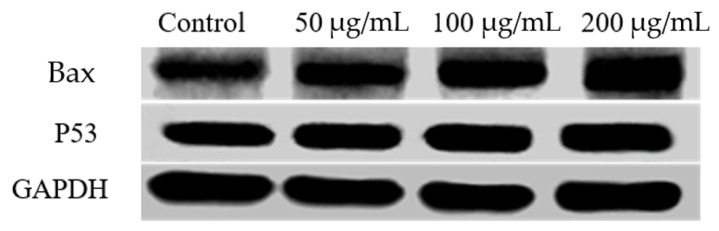
The effect of RLE on the expression level of apoptosis-associated protein in HT-29 cell.

Since the corresponding genes of Bax and P53 would be significantly upregulated in the RLE treatment group, Bax and P53 were selected for further western blotting analysis. HT-29 cells were firstly treated with 200, 100, 50, and 0 μg/mL concentration RLE for 48 h. Western blot results, depicted in Figure 9, showed that the expression level of Bax protein was significantly increased and the expression of P53 was also enhanced in the HT-29 cells treated with RLE, compared with the control group. Moreover, with the increase in the concentration, the expression difference was more and more significantly in a concentration-dependent upregulation, which was basically consistent with the results of qRT-PCR.

### 2.7. Analysis of Anticancer Active Components in RLE by GC-MS

**Figure 10:**
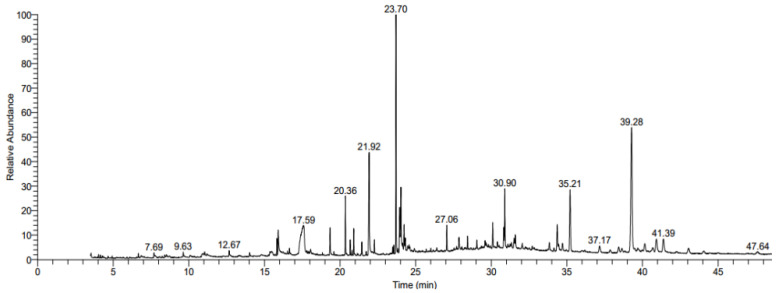
The total ion chromatograms of compounds determined by GC-MS of RLE.

The result of GC-MS analysis indicated that 31 compounds were detected in RLE (Figure 10). It mainly consists of seven components: alcohols (16.92%), phytosterols (21.25%), alkanes and alkenes (7.37%), fatty acids (17.31%), esters (4.59%), phenolic compounds (9.61%), and minor groups (0.49%), in which β-sitosterol was the highest abundance component (16.13%), and vitamin E, phytol, 3,7,11,15-tetramethyl-2-hexadecen-1-ol, squalene, palmitic acid, and α-linolenic acid were also detected at abundance, higher than 3%. Among them, there are 17 compounds with an anticancer effect, as shown in Supplementary Materials (Table S1); the results show that *R. molle* has potential anticancer value.

## 3. Discussion

As a traditional Chinese medicine, *R. molle* has been widely used in the prevention and treatment of various diseases for a long time. The roots, flowers, and fruits of *R. molle* have been commonly recorded in ancient medical monographs as traditional herbal medicines in China for analgesics, anti-inflammatory, anesthetic, sedative, insecticides, etc. [15,16]. In recent years, as a result of excessive logging and excavation, the resources of *R. molle* have been sharply decreased. The root of *R. molle* is nonrenewable, and long-term excavation will cause a devastating disaster to the medicinal plant resources of *R. molle*, which is not conducive to sustainable development. In addition, the flower and fruit collection of *R. molle* were affected by season. The leaves of *R. molle* are renewable, large in quantity, and easy to obtain, which are rich resources for the research of *R. molle*. In this study, we found that *R. molle* leaf has stronger anti-cancer ability than the flower, and can block HT-29 cells in S phase to promote cell apoptosis. These results indicated that the leaves of *R. molle* also have great medicinal value, and it might provide a substitute for the roots, flowers, and fruits of *R. molle*, and also offers a potential treatment option for colorectal cancer.

The normal position proliferation of cancer cells is essential for their carcinogenicity and invasiveness. It relies on the unmonitored rapid mitosis, and drugs targeting the mitosis process to inhibit the proliferation of cancer cells have been a popular area of traditional anticancer research. In this study, the growth inhibition of RLE in HT-29 cells is dose dependent. The cell viability of HT-29 cells was decreased from 76.09% to 18.35% with various concentrations of RLE (25–200 μg/mL) for 72 h, respectively. Furthermore, the prolonged inhibition of RLE on cells was observed by colony formation assay, suggesting that an irreversible signal of cell growth inhibition was achieved. Therefore, RLE could significantly inhibit the growth of HT-29 cells.

Tumor metastasis is an important pathological characteristic of a malignant tumor, which is the main cause of clinical treatment failure and high mortality [21]. Therefore, looking for drugs to prevent cancer metastasis is a breakthrough point in cancer treatment. The migration ability of cancer cells is the key to measure cancer metastasis, which is the key factor to determine the malignant degree of a tumor [22]. In this study, we found that the migration capacity of HT-29 cells in vitro was decreased with the increasing RLE concentration. The results showed that RLE could significantly inhibit the migration of colorectal cancer HT-29 cells. Compared with many anticancer drugs that inhibit the migration of cancer cells [22], RLE has the advantages of having less toxic side effects and being easy to obtain, so it is a potential anticancer traditional Chinese medicine with research value.

Different from necrosis, apoptosis is an important cellular death mechanism that does not generate an indirect destructive inflammatory response to normal cells in the surrounding microenvironment [21,23]. Thus, apoptosis is a protective mechanism that maintains tissue homeostasis by removing diseased cells [23,24]. Promoting apoptosis can inhibit the growth of tumor cells, thus inhibiting the occurrence and development of a tumor; inducing apoptosis of tumor cells is an important method to fight cancer [25]. However, cancer cells exhibit resistance to apoptosis to maintain their uncontrolled proliferation [26,27]. Hence, any compound with apoptosis regulation activity is likely to become a potential anticancer chemotherapy agent [28]. In this study, DAPI staining and flow cytometry were used to study the effect of RLE on apoptosis of colorectal cancer HT-29 cells. The nuclei of HT-29 cells treated with RLE showed typical apoptotic characteristics. The proportion of late apoptotic cells treated with different concentration of RLE was higher than that of the control group, and the inhibition degree increased as the concentration grew, thus indicating that RLE could bring HT-29 cell apoptosis. Promoting apoptosis is the way that many natural products play an anticancer role, and *R. molle* has a lower effective drug concentration compared with other Chinese herbal medicines [11,29].

The cell cycle is an important biological activity, which can regulate the proliferation of normal cells. However, in tumor cells, the cell cycle becomes abnormal or out of control [30]. Programmed cell death or apoptosis is an effective way to terminate normal cells and reuse energy. The apoptotic pathway in cancer cells is often greatly changed to break the cell cycle, resulting in an abnormal increase of cell life and function [31]. Under this background, the potential of cell cycle regulators and natural cell cycle regulation drugs in cancer treatment has been of interest for society [11]. Our results of cell cycle detection based on flow cytometry showed that HT-29 cells treated with RLE would block the cancer cells from going into S stage by destroying the cell cycle or inducing apoptosis, which achieved an anti-tumor effect. This is similar to the inhibitory mechanism of crude methanol extract of *Echinophora platyloba* on human breast cancer cells, but the effect is much stronger and requires a smaller concentration [11].

Previous studies indicated that uncontrolled cell division depends on the activation of cyclins, which bind to cyclin-dependent kinases (CDK) to induce cell cycle progression towards the S phase [11]. CDK activity is one of the major factors related to cancer deterioration. Their functions are rigorously regulated by related proteins, cyclin-dependent kinase inhibitors (CDKI), such as P21 protein [32]. P21 is a universal inhibitor of CDK(s) capable of binding with nearly all cyclin-CDK complexes, and thus inhibiting protein kinase activity and causing cell cycle block [33]. *P53* is a key intracellular tumor suppressor gene, which plays an important role in regulating cell proliferation, differentiation, and apoptosis, as well as *P21* gene expression [34]. Studies have revealed that Bcl-2 family protein plays a vital regulatory role in apoptosis as an activator (Bax) or inhibitor (Bcl-2) [35]. The expression level of Bax/Bcl-2, which belongs to the Bcl-2 protein family, is crucial to cell survival or apoptosis [36,37]. In cells, when the expression level of Bax was high, Bcl-2 and Bax formed homodimer Bcl-2/Bax through a phosphate diester dehydrogenation bond to promote cell apoptosis, while, when the expression level of Bcl-2 was high, heterodimer Bcl-2/Bax was formed through a hydrogen bond to inhibit cell apoptosis [38,39]. In this study, qRT-PCR and the western blot method were adopted to detect the expression of apoptosis-related genes and protein, so as to investigate the mechanism of HT-29 cell apoptosis mediated by RLE. The qPCR analysis showed that RLE could upregulate the expression of *Bax*, *P21*, and *P53* in HT-29 cells, and downregulate the expression of *Bcl-2*; western blot analysis showed that RLE can upregulate the expression of Bax protein and P53 protein, which was basically consistent with the results of qRT-PCR. The results revealed that RLE can augment the expression of *P21* mRNA by increasing *P53* mRNA expression and eventually blocking the HT-29 cell at S phase, inhibiting proliferation, and RLE could induce apoptosis through a Bax/Bcl-2 mediated pathway.

GC-MS showed that there were a lot of active compounds in the leaves of *R. molle*, which had antioxidant, anticancer, anti-inflammatory, and antibacterial effects, indicating that *R. molle* had great medicinal value. Among them, more than half of the components have an anticancer effect (Table 1). 4-vinyl-2-methoxyphenol and β-amyrin have a significant inhibitory effect on colorectal cancer HT-29 cells [40,41]. This provides a theoretical basis for *R. molle* as a potential anticancer drug.

## 4. Experimental Section

### 4.1. Reagents and Chemicals

The A549, HT-29, A2780, SGC7901, and PC3 cell lines were obtained from the cell bank of Jiangxi University of Traditional Chinese Medicine. Dulbecco’s modified eagle medium (DMEM), fetal bovine serum (FBS), phosphate buffered solution (PBS), penicillin–streptomycin solution, thiazolyl blue tetrazolium bromide (MTT), TRIzol, and SYBR Premix Ex Taq II were purchased from TAKARA (Beijing, China). Antibodies of Bax, P53, and GAPDH were purchased from SERVICE BIO (Wuhan, China). All of the reagents and chemicals used in this study were analytical grade.

### 4.2. Cell Culture

A549, HT-29, A2780, SGC7901, and PC3 cell lines were cultured in DMEM medium, containing 10% fetal bovine serum and 1% penicillin/streptomycin, and cultivated in the incubator at 37 °C with 5% CO_2_. After passages, cells were incubated with RLE and applied for different experiments.

### 4.3. Sample Collection and Preparation of Extracts

The leaf and flower of *R. molle* were collected in July 2019 at Lushan Botanical Garden, Jiangxi Province, China. Following this, the samples were freeze-dried and stored at −20 °C in the herbarium of Jiangxi Normal University, numbered F-00215-00056 and F-00215-000040. The samples were authenticated by Prof. MY Wang, a taxonomist of Jiangxi Normal University.

Briefly, dried leaf and flower were pulverized and extracted with 75% ethanol (1:10, *w*/*v*) for approximately 1 h, and this was repeated 3 times. The leaf extract (RLE) and flower extract (RFE) were freezd-dried. Then, the RLE and RFE were dissolved in DMSO and stored at −20 °C.

### 4.4. MTT Assay

The cells of the logarithmic growth phase were maintained in the 96-well plate at a cell density of 3 × 10^3^/well. The 96-well plate was cultured in a 37 °C, 5% CO_2_ incubator. After 24 h, the medium was replaced with a complete medium 200 μL, containing RLE and RFE 400, 200, 100, 50, 25 μg/mL, and the control group was provided with an equal amount of DMSO complete medium. Each group was set in triplicate. After 72 h, the drug in the medium was removed. They were washed with PBS and 10 μL MTT solution (5 mg/mL, dissolved in PBS) and 90 μL medium were added to each well. They were incubated at 37 °C, 5% CO_2_ for 4 h. Then, the liquid in the 96-well plate removed with using a pipette and 200 μL DMSO were added to each well before incubating for 30 min. The optical density (A) was measured at 570 nm with a microplate reader. The experiment was repeated 3 times.

### 4.5. Colony Formation Assay

The cells of the logarithmic growth phase were maintained in the 6-well plate at a cell density of 700/well. Following a 24 h incubation, the medium was replaced with a complete medium 200 μL, containing RLE 200, 100, 50, 0 μg/mL and the cells were cultured in the incubator at 37 °C, 5% CO_2_ for 13 d. Following this, the medium was discarded. They were then washed with PBS, fixed with 4% paraformaldehyde, and stained with crystal violet. The number of clones were counted under a low power lens and the cloning efficiency was calculated.

### 4.6. Wound Healing Assay

The cells of the logarithmic growth phase were maintained in the 6-well plate at a cell density of 2 × 10^5^/well. They were incubated until the cells covered the well. Using the tips of a T-20 pipette, “#”s were drawn vertically in equal size. Three points on the “#” were randomly selected as the observation point of measurement. The cells were cultured with a medium containing *R. molle* extracts 100, 50, 25, 0 μg/mL and changes in the width of the scratches were recorded at 0, 24 and 48 h.

### 4.7. Detection of Apoptosis by DAPI Staining

The cells of the logarithmic growth phase were maintained in the 24-well plate at a density of 2 × 10^4^/well for 24 h. The medium was replaced with a complete medium 200 μL, containing RLE 400, 200, 100, 0 μg/mL, and the control group was provided with an equal amount of DMSO (0.1%) complete medium. They were incubated for 24 h in the incubator at 37 °C, 5% CO_2_. The liquid in the plate was removed using a pipette and the cells were washed with PBS twice. DAPI staining solution was added and they were incubated for 20 min at room temperature, avoiding light. They were then observed under a fluorescence microscope.

### 4.8. Detection of Apoptosis and Cell Cycle by PI Staining

The cells of the logarithmic growth phase were maintained in the 24-well plate at a density of 2 × 10^4^/well for 24 h. The cultured medium was replaced with a complete medium 200 μL, containing RLE 200, 100, 50, 0 μg/mL, aand the control group was provided with an equal amount of DMSO (0.1%) complete medium. These were incubated for 24 h. A single cell suspension was made and then centrifuged to collect the cells. Pre-cooled 70%, cold at 4 °C, ethanol was added to fix the cells for at least 18 h. Before the detection, all ethanol was removed and the cells were washed with PBS three times. They were then incubated for 30 min at 37 °C before flow cytometric analysis.

### 4.9. qRT-PCR Analysis

Pre-treatment of the cells was the same as the method in Section 2.4. Total cellular RNA was extracted with TRIzol reagent (Invitrogen, Carlsbad, CA, USA) according to the manufacturer’s instructions. The primers are listed in Table 2. The target gene was quantitatively analyzed by ABI fluorescence quantitative kit. The reaction procedure was: pre-degeneration at 95 °C for 5 min; then 95 °C, 30 s; 60 °C, 15 s for 40 cycles. Each group was made in triplicate. The reactions were setup in duplicates in 20 µL containing 10 µL of SYBR^®^ Premix Ex Taq II, 0.4 µL of ROX Reference Dye, 0.8 µL of each primer, 1 µL of cDNA template, and 6 µL of H_2_O. Each sample was tested three times. The relative level of the target gene was calculated with 2^−ΔΔCt^ and β-actin as the internal control gene.

### 4.10. Western Blot Analysis

The cells were cultured with 200, 100, 50, 0 μg/mL concentration of RLE for 48 h, removing the original cultured medium and washing cells twice with 1 mL PBS. Then, cells were collected and lysed using lysis buffer. The BCA quantitative kit was used to detect the protein concentration. The protein was mixed with the SDS loading buffer solution in a certain proportion and bathed at 97 °C for 7 min. A 12% separation glue and 4% concentrate glue were prepared for electrophoresis, and film was transfered and sealed. The primary antibody was added and incubated overnight. This was then rinsed four times with TBS. It was incubated in the secondary antibody in a shaking table at 4 °C for 1 h, and washed four times with TBS. After ECL development, the recording strip was shot.

### 4.11. Analysis of Components in RLE by GC-MS

The chemical composition of the RLE was measured using Thermo’s GC-MS system. This GC-MS system is fitted with a DB-5MS and coupled with a quadrupole mass detector. The oven temperature was programmed for 60 °C for 1 min, with an increase of 8 °C/min to 285 °C for 18 min. The mass scan range was 50 to 650 *m*/*z*. Individual compounds were identified by comparing fragmentation patterns in the mass spectra with those from the software database.

### 4.12. Statistical Analysis

Statistical analysis was carried out by SPSS statistical software. The *T* test was used for the analysis between two groups, whereas the single factor variance analysis was used for intergroup analysis.

## 5. Conclusions

This study indicated that RLE could inhibit proliferation, induce the apoptotic mechanism, and block up cancer cells going into the S phase for colorectal cancer HT-29 cells. The molecular events have identified that the efficacy of RLE was associated with the upregulation of *Bax* and *P53*, and *P21*, and downregulation of *Bcl-2* genes. This is the first report revealing the inhibition role of RLE on colorectal cancer. In addition, this study provided some useful data for further study on the potential mechanism of anticancer activity of *R. molle*, and laid a foundation for developing a potential anticancer drug.

## Figures and Tables

**Table 1:** The IC_50_ value of RLE and RFE on five kinds of cytotoxicity.

Sample	IC_50_ (μm/mL)				
	HT-29	A549	A2780	SGC-7901	PC3
RLE	73.08	115.59	120.29	194.22	307.43
RFE	552.86	812.68	634.61	1287.15	881.96

**Table 2:** Information of *qRT-PCR* primer.

	Forward Primer (5′ → 3′)	Reverse Primer (5′ → 3′)
*Bax*	GAGAGGTCTTTTTCCGAGTG	GGTGAGGAGGCTTGAGGAGT
*Bcl-2*	GCTACCTAAGAAAAACCTGG	CAAGAAACAAGGTCAAAGGG
*p53*	CTTTGAGGTGCGTGTTT	CAGTGCTCGCTTAGTGC
*p21*	GACACCACTGGAGGGTGACT	CAGGTCCACATGGTCTTCCT
*β-actin*	TGGCACCACACCTTCTACAAT	AGAGGCGTACAGGGATAGCAC

## Data Availability

Data are contained within the article and the Supplementary Materials.

## Supplementary Materials

The following are available online, Table S1: Potential anticancer compounds of the *R. molle* leaves.

## Sample Availability

Samples of the methanol extract of *R. molle* leaves and flowers are available from the authors.
