# Supplementary material for: Rhododendron molle G. Don Extract Induces Apoptosis and Inhibits Migration in Human Colorectal Cancer Cells and Potential Anticancer Components Analysis
Source: Molecules. 2021 May 18;26(10):2990. doi: 10.3390/molecules26102990 (PMC8157555; doi:10.3390/molecules26102990)
Supplement: Supplementary file 1 [file molecules-26-02990-s001.zip › molecules-1212798-supplementary.pdf]

Supplementary Materials

Supplement Table S1. Potential anticancer compounds of the *R. molle* leaves.

NO.	Retention time	Compound	Molecular formula	Area %
1	12.67	2-Methoxy-4-vinylphenol	$C_9H_{10}O_2$	0.51
2	15.83	Phenol, 2,4-bis(1,1-dimethylethyl) acid	$C_{14}H_{22}O$	0.66
3	19.34	tetradecanoic acid	$C_{14}H_{28}O_2$	1.61
4	21.44	Hexadecanoic acid, methyl ester	$C_{17}H_{34}O_2$	0.76
5	21.92	Palmitic acid	$C_{16}H_{32}O_2$	7.28
6	22.27	Hexadecanoic acid, ethyl ester	$C_{18}H_{36}O_2$	0.61
7	23.7	Phytol	$C_{20}H_{40}O$	11.61
8	23.94	cis-9, cis-12-Octadecadienoic acid	$C_{18}H_{32}O_2$	2.64
9	24.03	α-linolenic acid	$C_{18}H_{30}O_2$	4.52
10	24.23	Stearic acid	$C_{18}H_{36}O_2$	1.26
11	24.32	Ethyl 9,12,15-octadecatrienoate	$C_{20}H_{34}O_2$	0.66
12	30.9	squalene	$C_{30}H_{50}$	3.31
13	35.21	Vitamin E	$C_{29}H_{50}O_2$	6.19
14	37.17	Campesterol	$C_{28}H_{48}O$	0.84
15	39.28	Stigmast-5-en-3-ol, (3á,24S)	$C_{29}H_{50}O$	16.13
16	40.16	β-amyrin	$C_{30}H_{50}O$	1.03
17	41.39	α-amyrin	$C_{30}H_{50}O$	1.79
